# Beyond Chaperoning: The Multifaceted Role of FACT in Chromatin Transactions

**DOI:** 10.3390/ijms26115176

**Published:** 2025-05-28

**Authors:** Olesya Volokh, Vasily M. Studitsky, Olga S. Sokolova

**Affiliations:** 1 Faculty of Biology, Moscow Lomonosov University, 119234 Moscow, Russia; olesyavolokh@gmail.com; 2 Fox Chase Cancer Center, Philadelphia, PA 19111, USA; vasily.studitsky@fccc.edu

**Keywords:** FACT, RNAP, transcription

## Abstract

Eukaryotic transcription involves a complex interplay of protein factors that dynamically engage with chromatin at distinct stages. Among these, the histone chaperone FACT (Facilitates Chromatin Transcription) plays a unique role in nucleosome disassembly and reassembly during transcription, replication, and repair. While its functional importance is well established, the underlying structural mechanisms involved in these activities remain incompletely understood. The remarkable functional versatility of FACT in regulating genetic information processing likely stems from its distinctive structural and mechanical properties. This review focuses on the structural organization of FACT and analysis of the mechanisms involved in chromatin reorganization by this unusual histone chaperone.

## 1. Introduction

Eukaryotic chromatin represents a highly organized yet dynamic structure in which DNA is wrapped around histone octamers to form repeating nucleosomal units [1]. These nucleosomal structures not only enable genome compaction but also play a pivotal role in regulating essential cellular processes, including transcription, DNA replication, and repair [2] Access to genetic information by the transcriptional machinery is tightly controlled through intricate chromatin remodeling mechanisms involving both covalent histone modifications and specialized protein complexes [3].

Among the factors governing chromatin dynamics, the histone chaperone FACT (Facilitates Chromatin Transcription) stands out as a key regulator. Initially identified as a factor enabling RNA polymerase II to overcome nucleosomal barriers [4], this highly conserved complex exhibits a unique mode of action. Unlike ATP-dependent remodeling complexes, FACT functions as a specialized chaperone helping RNA polymerase II to partially dissociate H2A-H2B dimers without complete nucleosome disassembly during transcription [5]. Subsequent studies have revealed its involvement in diverse nuclear processes—from facilitating replication fork progression [6] to coordinating double-strand break repair [7]. The multifunctionality of FACT is reflected in the proposed expansion of its name to “Facilitates Chromatin Transactions” [8], emphasizing its central role in chromatin-associated processes.

Recent structural insights [9] have elucidated the molecular basis of FACT-mediated nucleosome reorganization, yet some key questions remain unresolved. In particular, how a single complex participates in such diverse cellular processes while maintaining functional specificity requires further investigation. This review focuses on our current understanding of the structural mechanisms underlying FACT-dependent nucleosome reorganization, with special emphasis on its conformational dynamics and interactions with chromatin components.

## 2. Structural Organization of the hFACT Chaperone

**Figure 1:**
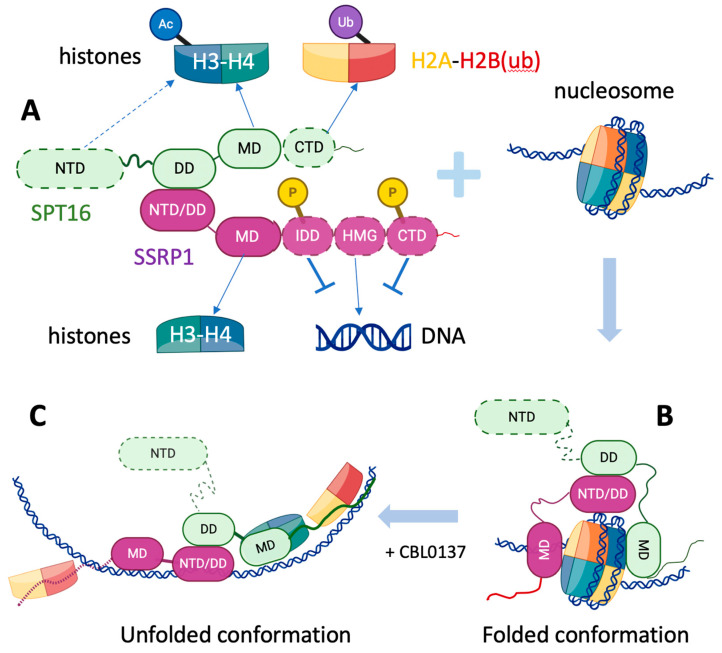
Organization and functioning of human FACT. (**A**) Domain organization. NTD—N-terminal domain, DD—dimerization domain, MD—middle domain, CTD—C-terminal intrinsically disordered domain, IDD—intrinsically disordered domain, HMG—HMG box domain; P—phosphorylation sites. Phosphorylation of SSRP1 reduces binding to DNA; Ac—histone acetylation; Ub—ubiquitinated H2B (H2Bub). The dashed lines and semi-transparent objects indicate mobile domains. (**B**) FACT interacts with intact nucleosomes. Upon partial uncoiling of nucleosomal DNA from the histone octamer in the presence of CBL0137, an unfolded conformation is formed (**C**). Built with biorender.com.

The hFACT complex represents an evolutionarily conserved heterodimeric assembly that exhibits remarkable structural plasticity during nucleosome interactions (Figure 1A). In mammalian systems, FACT comprises two principal subunits: SPT16 (Suppressor of Ty 16) and SSRP1 (Structure Specific Recognition Protein 1), forming a stable complex with an approximate molecular weight of 220 kDa [10,11]. SPT16 exhibits structural conservation across eukaryotes, including yeast and humans. This multifunctional protein contains four distinct domains: an N-terminal domain (NTD) that mediates interactions with H3-H4 histones [12]; a dimerization domain (DD) responsible for SSRP1 binding; a middle domain (MD) involved in DNA contacts; and a C-terminal domain (CTD) that stabilizes chromatin association [13].

In contrast to SPT16, the SSRP1 subunit displays significant interspecies variability. Mammalian SSRP1 contains a high-mobility group (HMG) domain that specifically recognizes and bends DNA, as demonstrated by atomic force microscopy studies [9]. Notably, in *Saccharomyces cerevisiae*, this function is compensated for by the independent Nhp6 protein, which contains an HMG-like motif and functionally substitutes for the absent HMG domain in the yeast Pob3 subunit [14]. Cryo-EM structural analyses have revealed that SSRP1 facilitates nucleosome reorganization through its intrinsically disordered domain (IDD), creating conditions for transient H2A-H2B dimer displacement while maintaining overall nucleosome integrity [5]. This modular architecture enables FACT to adapt to diverse chromatin conformational states, supporting its critical role in chromatin reorganization during essential nuclear processes.

Transmission electron microscopy analysis reveals human FACT as a modular, four-domain architecture adopting dynamic “closed” and “open” conformations [15]. In the compact state, full-length FACT exhibits a 5–6 nm globular density corresponding to SPT16-NTD—the largest structural module (~50 kDa)—positioned adjacent to three smaller domains (SSRP1-NTD/DD-SPT16-DD, SSRP1-MD, and SPT16-MD) of comparable mass (28–42 kDa). The truncation of SPT16-NTD eliminates this distinctive density while preserving the remaining tripartite organization, demonstrating its peripheral yet structurally autonomous positioning. These structural insights, detailed further in Section 4.3, demonstrate how SPT16-NTD mobility facilitates nucleosome engagement.

## 3. Physiological Functions of FACT

Extensive studies have established the FACT chaperone as a central regulator of chromatin dynamics. Biochemical and structural analyses demonstrate the unique ability of FACT to bind to all core histones, facilitating its involvement in essential nuclear processes [5]. During transcription, FACT promotes RNA polymerase II progression through nucleosomal barriers by transiently displacing H2A-H2B dimers while maintaining histone octamer integrity, as revealed by single-molecule FRET studies [8,16]. Genome-wide mapping approaches show the rapid recruitment of FACT to DNA damage sites, where it facilitates chromatin decompaction for repair machinery access [7]. Replication studies using *Xenopus* egg extracts demonstrate the cooperation of FACT with the MCM helicase complex in nucleosome disassembly ahead of replication forks [6]. These pleiotropic functions position FACT as a master coordinator of chromatin transactions, ensuring genome integrity while dynamically regulating DNA accessibility.

### 3.1. FACT in Transcription: Dynamic Regulation of Chromatin for Gene Expression

The FACT complex serves as a critical modulator of transcriptional processes through its unique ability to restructure chromatin architecture. Genome-wide localization studies demonstrate that FACT subunits exhibit preferential enrichment at the transcription start sites of active genes, where they facilitate the assembly of pre-initiation complexes by altering nucleosome positioning [17]. This reorganization of promoter-proximal chromatin creates accessible regions for transcription machinery binding, as evidenced by DNase I hypersensitivity assays [18].

During transcriptional elongation, FACT maintains a physical association with RNA polymerase II throughout transcribed regions [5]. Biochemical analyses reveal two distinct modes of FACT operation during this process. The first involves transient RNA polymerase II-driven displacement of H2A-H2B dimers to enable polymerase progression, supported by crosslinking studies showing the reduced stability of histone-DNA contacts and increased DNA accessibility at nucleosomal entry/exit sites in FACT-depleted systems [19]. The second mode features FACT-mediated stabilization of partially unwrapped nucleosomes without complete dimer removal (Figure 1C), as observed in single-molecule experiments [20,21]. These apparently contradictory mechanisms may represent context-dependent responses to different nucleosomal barriers.

The functional consequences of FACT activity extend beyond transcription facilitation. Quantitative chromatin immunoprecipitation experiments demonstrate that FACT is essential for maintaining nucleosome positioning and histone composition following transcriptional events [22]. This preservation of chromatin integrity is particularly crucial at highly expressed loci, where FACT prevents complete nucleosome loss [23]. Furthermore, FACT collaborates with chromatin remodelers such as SWI/SNF to activate transcription at stress-responsive genes, suggesting a coordinated mechanism for rapid gene induction [24].

While FACT is most extensively characterized in RNA polymerase II (RNAP II)-mediated transcription, it also plays roles in RNA polymerase I (Pol I) and III (Pol III) systems, facilitating ribosomal RNA and tRNA synthesis, respectively [25]. The distinct effects of SSRP1 versus SPT16 depletion on gene expression profiles further suggest subunit-specific regulatory functions. For example, Garcia et al. [26] demonstrated that SSRP1 knockdown preferentially disrupts the expression of pro-proliferative and oncogenic genes (e.g., *MYC*, *CCND1*), while SPT16 depletion predominantly affects chromatin organization and DNA repair pathways. This functional divergence—where SSRP1 modulates transcriptional programs linked to cell growth and transformation, whereas SPT16 maintains chromatin integrity—underscores the complexity of FACT’s involvement in transcriptional regulation across polymerase systems.

### 3.2. FACT Complex in DNA Replication: Maintaining Chromatin Integrity

The FACT complex plays an indispensable role in DNA replication by maintaining chromatin integrity during fork progression. Genetic studies in Saccharomyces cerevisiae first revealed the essential nature of the FACT subunits Pob3 and Spt16, demonstrating their physical interaction with DNA polymerase α and their requirement for both DNA synthesis and transcription [27,28,29]. Subsequent work in metazoans established that FACT associates with active replication forks, where it facilitates nucleosome disassembly ahead of the fork and promotes rapid reassembly behind it, ensuring chromatin survival during DNA synthesis [30,31]. This replication-coupled nucleosome recycling is critical for preserving epigenetic information during S phase. Mechanistically, FACT collaborates with the MCM helicase complex to unwind chromatinized DNA [32], while its interaction with the replisome ensures the proper processing of parental histones [33]. The FACT complex maintains normal replication fork progression by mitigating nucleosomal barriers and facilitating chromatin reorganization, thereby reducing indirect sources of torsional stress. This process works in concert with topoisomerases, which directly resolve DNA supercoiling. The depletion of FACT leads to fork stalling and genomic instability [6,34], likely due to unaddressed chromatin compaction hindering fork movement, even in the presence of functional topoisomerases.

Post-translational modifications regulate FACT replication functions. Ubiquitination by the cullin-E3 ligase Rtt101 targets FACT to replication forks [35], while its cooperation with the deubiquitinase Ubp10 modulates H2B ubiquitination dynamics during chromatin reassembly. These modifications create a precise spatiotemporal control system ensuring FACT activity is coordinated with fork progression. Notably, FACT shows differential interactions with leading and lagging strand machinery [35], suggesting specialized roles in handling the distinct chromatin challenges posed by each replicative strand.

The dual functionality of FACT in both transcription and replication creates potential conflicts during the S phase. Cells resolve this through the tight regulation of FACT recruitment, with replication taking precedence when forks encounter transcribed regions [36,37]. This prioritization reflects the essential nature of FACT replicative functions; while transcription requires chromatin opening, replication demands the complete disassembly and faithful restoration of nucleosomal arrays. The complex thus acts as a central coordinator of chromatin dynamics during genome duplication, bridging the gap between the DNA synthesis machinery and the epigenetic landscape [30,31]. Future studies using engineered replication systems should help to elucidate how multiple activities of FACT are integrated during fork progression and how its dysfunction contributes to replication stress-related diseases.

### 3.3. FACT Complex and Post-Translational Modifications: A Dynamic Regulatory Network

The functional versatility of the FACT complex in chromatin transactions is critically regulated through post-translational modifications (PTMs) of both its subunits and associated histones (schematic on Figure 1A). The phosphorylation of SSRP1 by casein kinase 2 (CK2) serves as a molecular switch, reducing its DNA-binding affinity while promoting recruitment to damaged chromatin [38]. This modification exemplifies how PTMs can spatially and temporally control FACT activity, with CK2-mediated phosphorylation being particularly important during the DNA damage response. Concurrently, FACT interaction with modified histones creates a sophisticated regulatory layer. The complex shows preferential binding to ubiquitinated H2B (H2Bub), a modification that facilitates nucleosome reorganization during transcription and repair [39]. Structural studies reveal that the FACT middle domain specifically recognizes H2Bub through a conserved binding interface, enabling the targeted reorganization of modified nucleosomes [39].

The interplay between FACT and histone acetylation marks further expands this regulatory network. FACT demonstrates functional synergy with H3K56ac, a modification enriched at transcriptionally active loci and replication forks [13]. This interaction maintains the chromatin architecture independently of transcription, suggesting FACT acts as a molecular interpreter of acetylation signals [13]. Similarly, methylation marks influence FACT dynamics, with SETD2-mediated H3K36me3 coordinating FACT recruitment during transcriptional elongation [40]. The complex ability to “read” these modifications while preventing their scrambling during chromatin disassembly and reassembly is crucial for epigenetic memory [41]. Notably, recent work has identified a ubiquitin-dependent regulatory axis involving OTUD5 and UBR5 that controls FACT activity at damaged chromatin, adding another layer to the already complex PTM-mediated regulation of FACT [42,43].

The mechanistic implications of these PTM networks are profound. First, they establish FACT as a central node in the crosstalk between chromatin modifiers and remodelers. Second, they provide a means for the context-specific regulation of FACT chaperone activity. For instance, phosphorylation–dephosphorylation cycles may dictate whether FACT participates in transcription, replication, or repair processes [38]. Finally, these modifications likely contribute to the paradox of FACT being both a stabilizer and destabilizer of nucleosomes—a duality that may be resolved by considering the PTM status of both histones and FACT itself. Understanding this intricate regulatory landscape remains challenging but essential, as perturbations in these pathways are increasingly linked to disease states, particularly in cancer where both FACT overexpression and aberrant PTM patterns are frequently observed.

### 3.4. FACT Complex in DNA Damage Repair: Chromatin Reorganization for Genome Stability

The FACT complex plays a pivotal role in DNA damage repair by modulating chromatin dynamics at lesion sites. Its recruitment to DNA breaks is facilitated by the phosphorylation of SSRP1 [44] and PARylation-dependent histone eviction [45], creating accessible chromatin for repair machinery. FACT coordinates multiple repair pathways: in homologous recombination, it promotes RAD51 loading and end resection [46] while interacting with RNF20 to regulate H2B ubiquitination [47,48]; in excision repair, it assists lesion recognition through SSRP1-XPC interactions [49] and enhances base excision repair via chromatin reorganization [50].

A key feature of FACT function is its ability to preserve epigenetic information during repair. By stabilizing modified histones like γH2AX [7,51] and maintaining H2B ubiquitination patterns [47,48], FACT ensures accurate chromatin restoration post-repair. This epigenetic safeguarding is crucial for maintaining genomic stability and preventing aberrant gene expression.

Despite the recent progress, fundamental questions remain about how FACT activities are regulated in different chromatin contexts. Future studies combining advanced imaging with engineered FACT variants will be essential to unravel these mechanisms. Understanding FACT’s multifaceted role in DNA repair not only advances basic science but also opens new avenues for targeting genomic instability in disease.

## 4. FACT Complex in Chromatin Homeostasis

### 4.1. A Master Regulator of Nucleosome Dynamics

The FACT complex operates as a central architect of chromatin integrity, employing unique mechanisms to both destabilize and preserve nucleosome structure. Structural analyses (Section 4.3) demonstrate FACT’s ability to stabilize partially unwrapped nucleosomes, preserving epigenetic memory. [21]. This “controlled destabilization” enables FACT to perform its dual functions: facilitating transcriptional elongation while maintaining epigenetic memory.

At the single-nucleosome level, FACT exhibits remarkable mechanical flexibility. Through multiple weak but cooperative interactions with histones H2A-H2B and H3-H4, the complex can either stabilize nucleosomes against spontaneous unwrapping or promote targeted disassembly when required for DNA-templated processes [52,53]. This bidirectional control depends on the post-translational modifications of both histones and FACT subunits, creating a responsive system that adjusts chromatin accessibility according to cellular needs. The ability of the complex to tether displaced histones to partially disassembled nucleosomes [54] explains how FACT maintains nucleosome integrity during transient unwrapping events that accompany transcription and repair.

The physiological implications of these molecular activities are profound. By maintaining the balance between chromatin accessibility and stability, FACT ensures the proper regulation of DNA-dependent processes while preventing catastrophic nucleosome loss. Disruption of this equilibrium leads to either excessive chromatin compaction or uncontrolled histone eviction, both associated with genomic instability. These findings position FACT not merely as a chaperone, but as an active modulator of chromatin states that responds to and integrates various cellular signals to preserve nucleosome homeostasis [21,53].

### 4.2. Nucleosome Recognition and Reorganization by the FACT Complex

The interaction between FACT and nucleosomes constitutes a sophisticated, multi-step process that orchestrates dynamic chromatin reorganization. Structural studies (Section 2 and Section 4.3) reveal that the SPT16 and SSRP1 subunits establish extensive contacts with all components of a nucleosome—DNA, the (H3-H4)_2_ tetramer, and H2A-H2B dimers—including SSRP1-HMG-induced DNA bending (Figure 1A) [5,12]. Notably, the HMG domain of SSRP1 selectively binds to the DNA minor groove, inducing localized bending of the double helix that facilitates the subsequent engagement of SPT16 with the histone octamer [13]. Unlike passive nucleosome binders, FACT actively stabilizes transitional chromatin states, including “hexasomal” particles lacking one H2A-H2B dimer [9]. This sophisticated binding mechanism enables FACT to mediate controlled nucleosome destabilization without complete histone dissociation in an ATP-independent way—a distinct feature that sets it apart from canonical ATP-dependent remodelers [14]. Crucially, FACT’s capacity for reversible nucleosome disassembly and reassembly is indispensable for regulating DNA accessibility during transcription, replication, and repair [55], underscoring its pivotal role in nuclear processes.

### 4.3. Mechanisms of Nucleosome Unfolding by FACT: Structural Insights

Building on earlier cryo-EM findings (Section 2), recent work has refined our understanding of FACT-mediated nucleosome reorganization. Human FACT binds asymmetrically to nucleosomes, with SPT16-MD engaging H2A-H2B and SSRP1-HMG bending in DNA ~40 bp from the dyad [21]. This binding pattern creates torsional stress that destabilizes approximately one-third of the nucleosomal DNA while leaving the remaining structure intact.

The unfolding mechanism appears conserved yet adaptable across species. Comparative EM studies of yeast and human FACT demonstrate similar overall architectures but reveal species-specific differences in DNA interaction patterns [56]. Yeast FACT (yFACT) requires the auxiliary factor Nhp6 for efficient nucleosome engagement, while human FACT achieves comparable destabilization through intrinsic domains. Both systems generate partially unfolded intermediates (Figure 1B) where approximately 50 base pairs of DNA become detached from the histone core, as quantified by EM image analysis [56]. This partial unfolding might be a universal intermediate state during FACT-mediated chromatin reorganization.

Curaxins, small molecules that destabilize nucleosomes, provide important insights into the mechanism of nucleosome unfolding by FACT. High-resolution EM structures of FACT–nucleosome complexes treated with the curaxin CBLC137 show enhanced DNA unwrapping compared to untreated samples [15]. The drug appears to stabilize FACT in a conformation that promotes more extensive DNA detachment, particularly at the entry/exit sites. Biochemical assays demonstrate that curaxin treatment increases FACT affinity for nucleosomes by approximately 3-fold while reducing the energy barrier for unfolding by 40%, suggesting a cooperative binding mechanism [15]).

Three distinct stages of FACT-mediated unfolding emerge from these studies:

(1) Initial engagement through asymmetric contacts with histones and DNA;
(2) Local DNA distortion leading to partial unwrapping;
(3) Stabilization of a metastable unfolded state.

The process appears ATP-independent but requires precise coordination between FACT subunits. Mutational analysis identifies key residues in the SPT16 acidic pocket that are essential for maintaining the unfolded state [21]. The disruption of these interactions prevents complete nucleosome reorganization while preserving initial binding.

Notably, FACT maintains contact with displaced histones throughout the unfolding process (Figure 1C), differing fundamentally from ATP-dependent remodelers that completely eject histones [56]. This “histone-retention” mechanism may explain FACT’s ability to rapidly restore the nucleosome structure after polymerase II passage during transcription. The emerging model positions FACT as a molecular stabilizer of transient chromatin states rather than a conventional remodeling motor.

### 4.4. FACT Complex as a Chromatin Plasticity Modulator in Cell Identity Transitions (Cell Fate Determination)

The FACT complex contributes to cell identity regulation primarily through its role in maintaining chromatin plasticity during transcription and replication, rather than acting as a direct driver of pluripotency. While FACT facilitates nucleosome reorganization at pluripotency loci (e.g., enabling Oct4 binding [57]), its function is mechanistically distinct from core transcription factors like Oct4 or Sox2, which directly activate lineage-specific programs. Instead, FACT acts as a chromatin accessibility hub, supporting transcriptional and epigenetic remodeling during cell state transitions. For instance, during reprogramming, FACT collaborates with transcription factors by resolving nucleosomal barriers at pluripotency gene promoters, allowing pioneer factors to access DNA [57,58,59,60]. This indirect role is further evidenced by its dispensability in maintaining established pluripotent states [58] contrasting with the essential, continuous requirement for Oct4.

FACT’s involvement in DNA replication and repair also indirectly impacts cell identity. By preserving epigenetic information during the S phase [6,31] and ensuring chromatin restoration post-DNA damage [7,51], FACT safeguards genomic and epigenetic stability—a prerequisite for faithful cell fate transitions. Thus, while FACT does not directly specify lineage programs, instead, its multifaceted chromatin regulatory functions create a permissive environment for transcription factors to execute cell identity changes.

Beyond pluripotency, FACT orchestrates lineage-specific differentiation through selective chromatin reorganization. In osteoblast differentiation, SSRP1 regulates Wnt signaling by mediating β-catenin recruitment [61], while in macrophage development, FACT partners with TRIM33 to remodel distal regulatory elements [62]. These context-dependent functions demonstrate FACT’s versatility in interpreting differentiation signals through chromatin reconfiguration. The ability of FACT to interact with lineage-determining factors suggests that it acts as a chromatin adaptor that translates developmental cues into epigenetic changes.

Key questions remain regarding how post-translational modifications regulate FACT activity in different cellular contexts and how its various protein partnerships dictate specific differentiation outcomes. Addressing these questions could provide new insights into manipulating cell identity for regenerative medicine applications. The dual role of FACT in both maintaining and reshaping chromatin states positions it as a central player in cell fate transitions, making it a promising target for controlling cellular plasticity.

### 4.5. Perspectives and Unresolved Questions in FACT-Mediated Nucleosome Unfolding

The current understanding of FACT-mediated nucleosome reorganization presents several intriguing paradoxes that warrant further investigation. While cryo-EM studies have established the structural framework for partial DNA unwrapping [21], the precise energetic determinants of this process remain controversial. The observed 40% reduction in the unfolding energy barrier with curaxins [15] suggests cooperativity in FACT–nucleosome interactions, yet the molecular basis for this cooperativity lacks definitive explanation. Alternative models propose that FACT may function as a “molecular wedge” creating localized stress points [56], while others emphasize its role as a dynamic scaffold stabilizing multiple transitional states [23]. This dichotomy reflects deeper questions about whether the primary function of FACT involves the active destabilization versus passive stabilization of chromatin intermediates.

Species-specific differences between yeast and human FACT systems raise additional mechanistic questions. The strict Nhp6-dependence in yeast [56] contrasts with the autonomous activity in human FACT, suggesting either divergent solutions to similar structural challenges or fundamentally distinct biological requirements. Recent single-molecule studies hint that these differences may reflect varying demands for processivity versus precision in different transcriptional contexts. Furthermore, the physiological relevance of observed in vitro unfolding intermediates remains debated, particularly regarding whether the 50 bp DNA detachment [56] represents a universal functional state or an experimental artifact.

Key unresolved questions include the following:

(1) How does FACT coordinate with other chromatin remodelers in vivo given its unique histone-retention mechanism?
(2) Does partial unfolding represent the endpoint or intermediate in physiological contexts?
(3) How do post-translational modifications alter FACT unfolding energetics?

Comparative analysis of yeast and human FACT reveals functional differences that may reflect adaptation to distinct genomic architectures [29,56]. While yeast FACT strictly requires Nhp6 for activity [29], human FACT operates autonomously [6], suggesting either divergent mechanisms or context-specific regulation. Single-molecule studies reveal varying processivity—from sustained engagement at ribosomal genes to transient interactions at stress-responsive loci [63]—but the physiological relevance of the observed intermediates (e.g., 50 bp DNA detachment) remains to be substantiated with other methods.

## 5. Conclusions

The structural and mechanistic studies of FACT summarized in this review have fundamentally advanced our understanding of chromatin plasticity. Cryo-EM has been instrumental in defining FACT’s histone-retention mechanism (Section 2 and Section 4.3) [21]. This ATP-independent process is conserved yet adaptable across species [56], positioning FACT as a master regulator of chromatin accessibility during transcription, replication, and repair.

Three principal findings have emerged from these investigations:

First, FACT displays remarkable functional plasticity across different nuclear processes. During transcriptional elongation, it facilitates RNA polymerase II progression through transient H2A-H2B dimer displacement while preventing complete nucleosome dissociation [5]. At replication forks, it coordinates the transfer of parental histones to newly synthesized DNA, maintaining epigenetic information through cell division [6]. These activities all occur while preserving core histone modification patterns, as demonstrated by quantitative proteomic analyses [53].

Second, comparative studies between yeast and humans highlight functional differences, underscoring FACT’s adaptation to distinct biological contexts [6,29]. Evolutionary conclusions require the inclusion of outgroups (e.g., plants or Xenopus) for validation. The essential requirement for Nhp6 in yeast FACT contrasts sharply with the autonomous activity observed in mammalian systems [29]. This difference likely reflects adaptation to distinct genomic architectures and regulatory demands between unicellular and multicellular organisms.

Third, the sensitivity of the complex to small-molecule inhibitors has revealed potential therapeutic vulnerabilities. Curaxins and related compounds specifically disrupt FACT–chromatin interactions by competing for histone binding surfaces [55]. This pharmacological profile suggests that the targeted modulation of FACT activity may be achievable in clinical contexts.

Key unresolved questions regarding the mechanism of nucleosome reorganization by FACT include the following: the structural basis of its nucleosome recognition specificity, regulation by post-translational modifications, and functional interplay with other chromatin modifiers in vivo. Addressing these questions will require time-resolved structural approaches, single-molecule imaging in native chromatin contexts, and cellular systems with engineered FACT variants. Such studies should clarify how FACT maintains the crucial balance between chromatin accessibility and stability during nuclear processes, while preserving histone integrity—a unique capability among chromatin remodelers that continues to challenge our understanding of epigenetic regulation.

## Abbreviations

The following abbreviations are used in this manuscript:

FACT	Facilitates Chromatin Transcription
SPT16	Suppressor of Ty 16
SSRP1	Structure Specific Recognition Protein 1
HMG	High-mobility group
FRET	Fluorescence Resonance Energy Transfer
NTD	N-terminal domain
Cryo-EM	Cryoelectron microscopy
MCM	Minichromosome maintenance
PTM	Post-translational modifications
CK2	Casein kinase 2
RNAP	RNA polymerase

## References

[1] Luger K., Richmond R.K., Sargent D.F., Richmond T.J., Ma A.W. (1997). Crystal Structure of the Nucleosome Core Particle at 2.8 Å Resolution. Nature.

[2] Kornberg R.D., Lorch Y. (1999). Twenty-Five Years of the Nucleosome, Fundamental Particle of the Eukaryote Chromosome. Cell.

[3] Jenuwein T., Allis C.D. (2001). Translating the Histone Code. Science.

[4] Orphanides G., LeRoy G., Chang C.-H., Luse D.S., Reinberg D. (1998). FACT, a Factor That Facilitates Transcript Elongation Through Nucleosomes. Cell.

[5] Belotserkovskaya R., Oh S., Bondarenko V.A., Orphanides G., Studitsky V.M., Reinberg D. (2003). FACT Facilitates Transcription-Dependent Nucleosome Alteration. Science.

[6] Abe T., Sugimura K., Hosono Y., Takami Y., Akita M., Yoshimura A., Tada S., Nakayama T., Murofushi H., Okumura K. (2011). The Histone Chaperone Facilitates Chromatin Transcription (FACT) Protein Maintains Normal Replication Fork Rates. J. Biol. Chem..

[7] Heo K., Kim H., Choi S.H., Choi J., Kim K., Gu J., Lieber M.R., Yang A.S., An W. (2008). FACT-Mediated Exchange of Histone Variant H2AX Regulated by Phosphorylation of H2AX and ADP-Ribosylation of Spt16. Mol. Cell.

[8] Formosa T. (2008). FACT and the Reorganized Nucleosome. Mol. Biosyst..

[9] Hondele M., Stuwe T., Hassler M., Halbach F., Bowman A., Zhang E.T., Nijmeijer B., Kotthoff C., Rybin V., Amlacher S. (2013). Structural Basis of Histone H2A–H2B Recognition by the Essential Chaperone FACT. Nature.

[10] Brewster N.K., Johnston G.C., Singer R.A. (1998). Characterization of the CP Complex, an Abundant Dimer of Cdc68 and Pob3 Proteins That Regulates Yeast Transcriptional Activation and Chromatin Repression. J. Biol. Chem..

[11] Brewster N.K., Johnston G.C., Singer R.A. (2001). A Bipartite Yeast SSRP1 Analog Comprised of Pob3 and Nhp6 Proteins Modulates Transcription. Mol. Cell Biol..

[12] Kemble D.J., McCullough L.L., Whitby F.G., Formosa T., Hill C.P. (2015). FACT Disrupts Nucleosome Structure by Binding H2A-H2B with Conserved Peptide Motifs. Mol. Cell.

[13] McCullough L.L., Connell Z., Xin H., Studitsky V.M., Feofanov A.V., Valieva M.E., Formosa T. (2018). Functional Roles of the DNA-Binding HMGB Domain in the Histone Chaperone FACT in Nucleosome Reorganization. J. Biol. Chem..

[14] Formosa T. (2012). The Role of FACT in Making and Breaking Nucleosomes. Biochim. Biophys. Acta (BBA)-Gene Regul. Mech..

[15] Volokh O.I., Sivkina A.L., Moiseenko A.V., Popinako A.V., Karlova M.G., Valieva M.E., Kotova E.Y., Kirpichnikov M.P., Formosa T., Studitsky V.M. (2022). Mechanism of Curaxin-Dependent Nucleosome Unfolding by FACT. Front. Mol. Biosci..

[16] VanDemark A.P., Xin H., McCullough L., Rawlins R., Bentley S., Heroux A., Stillman D.J., Hill C.P., Formosa T. (2008). Structural and Functional Analysis of the Spt16p N-Terminal Domain Reveals Overlapping Roles of YFACT Subunits. J. Biol. Chem..

[17] Saunders A., Werner J., Andrulis E.D., Nakayama T., Hirose S., Reinberg D., Lis J.T. (2003). Tracking FACT and the RNA Polymerase II Elongation Complex Through Chromatin in Vivo. Science.

[18] Orphanides G., Wu W.-H., Lane W.S., Hampsey M., Reinberg D. (1999). The Chromatin-Specific Transcription Elongation Factor FACT Comprises Human SPT16 and SSRP1 Proteins. Nature.

[19] Xin H., Takahata S., Blanksma M., McCullough L., Stillman D.J., Formosa T. (2009). YFACT Induces Global Accessibility of Nucleosomal DNA Without H2A-H2B Displacement. Mol. Cell.

[20] Valieva M.E., Armeev G.A., Kudryashova K.S., Gerasimova N.S., Shaytan A.K., Kulaeva O.I., McCullough L.L., Formosa T., Georgiev P.G., Kirpichnikov M.P. (2016). Large-Scale ATP-Independent Nucleosome Unfolding by a Histone Chaperone. Nat. Struct. Mol. Biol..

[21] Liu Y., Zhou K., Zhang N., Wei H., Tan Y.Z., Zhang Z., Carragher B., Potter C.S., D’Arcy S., Luger K. (2020). FACT Caught in the Act of Manipulating the Nucleosome. Nature.

[22] Jeronimo C., Watanabe S., Kaplan C.D., Peterson C.L., Robert F. (2015). The Histone Chaperones FACT and Spt6 Restrict H2A.Z from Intragenic Locations. Mol. Cell.

[23] Formosa T., Winston F. (2020). The Role of FACT in Managing Chromatin: Disruption, Assembly, or Repair?. Nucleic Acids Res..

[24] Floer M., Wang X., Prabhu V., Berrozpe G., Narayan S., Spagna D., Alvarez D., Kendall J., Krasnitz A., Stepansky A. (2010). A RSC/Nucleosome Complex Determines Chromatin Architecture and Facilitates Activator Binding. Cell.

[25] Birch J.L., Tan B.C.-M., Panov K.I., Panova T.B., Andersen J.S., Owen-Hughes T.A., Russell J., Lee S.-C., Zomerdijk J.C.B.M. (2009). FACT Facilitates Chromatin Transcription by RNA Polymerases I and III. EMBO J..

[26] Garcia H., Miecznikowski J.C., Safina A., Commane M., Ruusulehto A., Kilpinen S., Leach R.W., Attwood K., Li Y., Degan S. (2013). Facilitates Chromatin Transcription Complex Is an “Accelerator” of Tumor Transformation and Potential Marker and Target of Aggressive Cancers. Cell Rep..

[27] Schlesinger M.B., Formosa T. (2000). *POB3* Is Required for Both Transcription and Replication in the Yeast *Saccharomyces cerevisiae*. Genetics.

[28] Wittmeyer J., Formosa T. (1997). The *Saccharomyces cerevisiae* DNA Polymerase α Catalytic Subunit Interacts with Cdc68/Spt16 and with Pob3, a Protein Similar to an HMG1-Like Protein. Mol. Cell Biol..

[29] Wittmeyer J., Joss L., Formosa T. (1999). Spt16 and Pob3 of *Saccharomyces cerevisiae* form an Essential, Abundant Heterodimer That Is Nuclear, Chromatin-Associated, and Copurifies with DNA Polymerase α. Biochemistry.

[30] Serra-Cardona A., Zhang Z. (2018). Replication-Coupled Nucleosome Assembly in the Passage of Epigenetic Information and Cell Identity. Trends Biochem. Sci..

[31] Yang J., Zhang X., Feng J., Leng H., Li S., Xiao J., Liu S., Xu Z., Xu J., Li D. (2016). The Histone Chaperone FACT Contributes to DNA Replication-Coupled Nucleosome Assembly. Cell Rep..

[32] Tan B.C.-M., Chien C.-T., Hirose S., Lee S.-C. (2006). Functional Cooperation Between FACT and MCM Helicase Facilitates Initiation of Chromatin DNA Replication. EMBO J..

[33] Foltman M., Evrin C., De Piccoli G., Jones R.C., Edmondson R.D., Katou Y., Nakato R., Shirahige K., Labib K. (2013). Eukaryotic Replisome Components Cooperate to Process Histones During Chromosome Replication. Cell Rep..

[34] Kurat C.F., Yeeles J.T.P., Patel H., Early A., Diffley J.F.X. (2017). Chromatin Controls DNA Replication Origin Selection, Lagging-Strand Synthesis, and Replication Fork Rates. Mol. Cell.

[35] Zhai Y., Li N., Jiang H., Huang X., Gao N., Tye B.K. (2017). Unique Roles of the Non-Identical MCM Subunits in DNA Replication Licensing. Mol. Cell.

[36] Okuhara K., Ohta K., Seo H., Shioda M., Yamada T., Tanaka Y., Dohmae N., Seyama Y., Shibata T., Murofushi H. (1999). A DNA Unwinding Factor Involved in DNA Replication in Cell-Free Extracts of Xenopus Eggs. Curr. Biol..

[37] Hertel L., De Andrea M., Bellomo G., Santoro P., Landolfo S., Gariglio M. (1999). The HMG Protein T160 Colocalizes with DNA Replication Foci and Is Down-Regulated During Cell Differentiation. Exp. Cell Res..

[38] Li Y., Keller D.M., Scott J.D., Lu H. (2005). CK2 Phosphorylates SSRP1 and Inhibits Its DNA-Binding Activity. J. Biol. Chem..

[39] Mayanagi K., Saikusa K., Miyazaki N., Akashi S., Iwasaki K., Nishimura Y., Morikawa K., Tsunaka Y. (2019). Structural Visualization of Key Steps in Nucleosome Reorganization by Human FACT. Sci. Rep..

[40] Carvalho S., Raposo A.C., Martins F.B., Grosso A.R., Sridhara S.C., Rino J., Carmo-Fonseca M., de Almeida S.F. (2013). Histone Methyltransferase SETD2 Coordinates FACT Recruitment with Nucleosome Dynamics During Transcription. Nucleic Acids Res..

[41] Jeronimo C., Poitras C., Robert F. (2019). Histone Recycling by FACT and Spt6 during Transcription Prevents the Scrambling of Histone Modifications. Cell Rep..

[42] de Vivo A., Sanchez A., Yegres J., Kim J., Emly S., Kee Y. (2019). The OTUD5-UBR5 Complex Regulates FACT-Mediated Transcription at Damaged Chromatin. Nucleic Acids Res..

[43] Sanchez A., De Vivo A., Uprety N., Kim J., Stevens S.M., Kee Y. (2016). BMI1–UBR5 Axis Regulates Transcriptional Repression at Damaged Chromatin. Proc. Natl. Acad. Sci. USA.

[44] Krohn N.M., Stemmer C., Fojan P., Grimm R., Grasser K.D. (2003). Protein Kinase CK2 Phosphorylates the High Mobility Group Domain Protein SSRP1, Inducing the Recognition of UV-Damaged DNA. J. Biol. Chem..

[45] Yang G., Chen Y., Wu J., Chen S.-H., Liu X., Singh A.K., Yu X. (2020). Poly(ADP-Ribosyl)Ation Mediates Early Phase Histone Eviction at DNA Lesions. Nucleic Acids Res..

[46] Kumari A., Mazina O.M., Shinde U., Mazin A.V., Lu H. (2009). A Role for SSRP1 in Recombination-mediated DNA Damage Response. J. Cell Biochem..

[47] Oliveira D.V., Kato A., Nakamura K., Ikura T., Okada M., Kobayashi J., Yanagihara H., Saito Y., Tauchi H., Komatsu K. (2013). Histone Chaperone FACT Regulates Homologous Recombination by Chromatin Remodeling Through Interaction with RNF20. J. Cell Sci..

[48] Nakamura K., Kato A., Kobayashi J., Yanagihara H., Sakamoto S., Oliveira D.V.N.P., Shimada M., Tauchi H., Suzuki H., Tashiro S. (2011). Regulation of Homologous Recombination by RNF20-Dependent H2B Ubiquitination. Mol. Cell.

[49] Wienholz F., Zhou D., Turkyilmaz Y., Schwertman P., Tresini M., Pines A., van Toorn M., Bezstarosti K., Demmers J.A.A., Marteijn J.A. (2019). FACT Subunit Spt16 Controls UVSSA Recruitment to Lesion-Stalled RNA Pol II and Stimulates TC-NER. Nucleic Acids Res..

[50] Charles Richard J.L., Shukla M.S., Menoni H., Ouararhni K., Lone I.N., Roulland Y., Papin C., Ben Simon E., Kundu T., Hamiche A. (2016). FACT Assists Base Excision Repair by Boosting the Remodeling Activity of RSC. PLoS Genet..

[51] Piquet S., Le Parc F., Bai S.-K., Chevallier O., Adam S., Polo S.E. (2018). The Histone Chaperone FACT Coordinates H2A.X-Dependent Signaling and Repair of DNA Damage. Mol. Cell.

[52] Winkler D.D., Luger K. (2011). The Histone Chaperone FACT: Structural Insights and Mechanisms for Nucleosome Reorganization. J. Biol. Chem..

[53] Chen P., Dong L., Hu M., Wang Y.-Z., Xiao X., Zhao Z., Yan J., Wang P.-Y., Reinberg D., Li M. (2018). Functions of FACT in Breaking the Nucleosome and Maintaining Its Integrity at the Single-Nucleosome Level. Mol. Cell.

[54] Wang T., Liu Y., Edwards G., Krzizike D., Scherman H., Luger K. (2018). The Histone Chaperone FACT Modulates Nucleosome Structure by Tethering Its Components. Life Sci. Alliance.

[55] Gurova K., Chang H.-W., Valieva M.E., Sandlesh P., Studitsky V.M. (2018). Structure and Function of the Histone Chaperone FACT—Resolving FACTual Issues. Biochim. Biophys. Acta (BBA)-Gene Regul. Mech..

[56] Sivkina A.L., Karlova M.G., Valieva M.E., McCullough L.L., Formosa T., Shaytan A.K., Feofanov A.V., Kirpichnikov M.P., Sokolova O.S., Studitsky V.M. (2022). Electron Microscopy Analysis of ATP-Independent Nucleosome Unfolding by FACT. Commun. Biol..

[57] Ding J., Xu H., Faiola F., Ma’ayan A., Wang J. (2012). Oct4 Links Multiple Epigenetic Pathways to the Pluripotency Network. Cell Res..

[58] Shen Z., Formosa T., Tantin D. (2018). FACT Inhibition Blocks Induction But Not Maintenance of Pluripotency. Stem Cells Dev..

[59] Garcia H., Fleyshman D., Kolesnikova K., Safina A., Commane M., Paszkiewicz G., Omelian A., Morrison C., Gurova K. (2011). Expression of FACT in Mammalian Tissues Suggests Its Role in Maintaining of Undifferentiated State of Cells. Oncotarget.

[60] Koche R.P., Smith Z.D., Adli M., Gu H., Ku M., Gnirke A., Bernstein B.E., Meissner A. (2011). Reprogramming Factor Expression Initiates Widespread Targeted Chromatin Remodeling. Cell Stem Cell.

[61] Hossan T., Nagarajan S., Baumgart S.J., Xie W., Magallanes R.T., Hernandez C., Chiaroni P.-M., Indenbirken D., Spitzner M., Thomas-Chollier M. (2016). Histone Chaperone SSRP1 Is Essential for Wnt Signaling Pathway Activity During Osteoblast Differentiation. Stem Cells.

[62] Ferri F., Petit V., Barroca V., Romeo P.-H. (2019). Interplay Between FACT Subunit SPT16 and TRIM33 Can Remodel Chromatin at Macrophage Distal Regulatory Elements. Epigenet. Chromatin.

[63] Martin B.J.E., Chruscicki A.T., Howe L.J. (2018). Transcription Promotes the Interaction of the FAcilitates Chromatin Transactions (FACT) Complex with Nucleosomes in *Saccharomyces cerevisiae*. Genetics.

